# Assessment of perfusion deficit with early phases of [^18^F]PI-2620 tau-PET versus [^18^F]flutemetamol-amyloid-PET recordings

**DOI:** 10.1007/s00259-022-06087-y

**Published:** 2022-12-27

**Authors:** Friederike Völter, Leonie Beyer, Florian Eckenweber, Maximilian Scheifele, Ngoc Bui, Marianne Patt, Henryk Barthel, Sabrina Katzdobler, Carla Palleis, Nicolai Franzmeier, Johannes Levin, Robert Perneczky, Boris-Stephan Rauchmann, Osama Sabri, Jimin Hong, Paul Cumming, Axel Rominger, Kuangyu Shi, Peter Bartenstein, Matthias Brendel

**Affiliations:** 1 Department of Nuclear Medicine, University Hospital of Munich, LMU Munich, Munich, Germany; 2 Department of Nuclear Medicine, University of Leipzig, Leipzig, Germany; 3 Department of Neurology, University Hospital of Munich, LMU Munich, Munich, Germany; 4 German Center for Neurodegenerative Diseases (DZNE), Munich, Germany; 5 Institute for Stroke and Dementia Research (ISD), Munich, Germany; 6 Munich Cluster for Systems Neurology (SyNergy), Munich, Germany; 7 Department of Psychiatry and Psychotherapy, University Hospital, LMU Munich, Munich, Germany; 8 Ageing Epidemiology (AGE) Research Unit, School of Public Health, Imperial College London, London, UK; 9 Sheffield Institute for Translational Neuroscience (SITraN), University of Sheffield, Sheffield, UK; 10 Department of Radiology, University Hospital, LMU Munich, Munich, Germany; 11 Department of Nuclear Medicine, Bern University Hospital, Bern, Switzerland; 12 School of Psychology and Counselling, Queensland University of Technology, Brisbane, Australia

**Keywords:** Neuroimaging, Perfusion phase, [^18^F]PI-2620, [^18^F]Flutemetamol

## Abstract

**Purpose:**

Characteristic features of amyloid-PET (A), tau-PET (T), and FDG-PET (N) can serve for the A/T/N classification of neurodegenerative diseases. Recent studies showed that the early, perfusion-weighted phases of amyloid- or tau-PET recordings serve to detect cerebrometabolic deficits equally to FDG-PET, therefore providing a surrogate of neuronal injury. As such, two channels of diagnostic information can be obtained in the setting of a single PET scan. However, there has hitherto been no comparison of early-phase amyloid- and tau-PET as surrogates for deficits in perfusion/metabolism. Therefore, we undertook to compare [^18^F]flutemetamol-amyloid-PET and [^18^F]PI-2620 tau-PET as “one-stop shop” dual purpose tracers for the detection of neurodegenerative disease.

**Methods:**

We obtained early-phase PET recordings with [^18^F]PI-2620 (0.5–2.5 min p.i.) and [^18^F]flutemetamol (0–10 min p.i.) in 64 patients with suspected neurodegenerative disease. We contrasted global mean normalized images (SUVr) in the patients with a normal cohort of 15 volunteers without evidence of increased pathology to β-amyloid- and tau-PET examinations. Regional group differences of tracer uptake (z-scores) of 246 Brainnetome volumes of interest were calculated for both tracers, and the correlations of the z-scores were evaluated using Pearson’s correlation coefficient. Lobar compartments, regions with significant neuronal injury (z-scores <  − 3), and patients with different neurodegenerative disease entities (e.g., Alzheimer’s disease or 4R-tauopathies) served for subgroup analysis. Additionally, we used partial regression to correlate regional perfusion alterations with clinical scores in cognition tests.

**Results:**

The z-scores of perfusion-weighted images of both tracers showed high correlations across the brain, especially in the frontal and parietal lobes, which were the brain regions with pronounced perfusion deficit in the patient group (*R* = 0.83 ± 0.08; range, 0.61–0.95). Z-scores of individual patients correlated well by region (*R* = 0.57 ± 0.15; range, 0.16–0.90), notably when significant perfusion deficits were present (*R* = 0.66 ± 0.15; range, 0.28–0.90).

**Conclusion:**

The early perfusion phases of [^18^F]PI-2620 tau- and [^18^F]flutemetamol-amyloid-PET are roughly equivalent indices of perfusion defect indicative of regional and lobar neuronal injury in patients with various neurodegenerative diseases. As such, either tracer may serve for two diagnostic channels by assessment of amyloid/tau status and neuronal activity.

**Supplementary Information:**

The online version contains supplementary material available at 10.1007/s00259-022-06087-y.

## Introduction

As our society is demographically aging, the incidence of neurodegenerative diseases is on the rise; the Dementia Forecasting Collaborators predict a 166% increase of the global prevalence of dementia in the coming three decades [1]. Treatments under development for neurodegenerative diseases such as Alzheimer’s disease (AD) include anti-β-amyloid-antibodies such as aducanumab and donanemab [2, 3]. A timely and precise diagnosis and therapy monitoring of objective endpoints is crucial for the development of successful therapies. Multiple studies have used FDG-PET/CT for the localization and quantification of cerebrometabolic defects indicative of neuronal injury during AD treatment trials [4–6]. Furthermore, aggregation of misfolded proteins leading to neuronal damage can be detected years before symptom onset with PET tracers for β-amyloid (Aβ) and tau. Assessments of neuronal injury, β-amyloid, and tau were recently embedded in the A/T/N scheme in the framework of AD research [7, 8]. This detailed assessment of patients by ATN indices may enable the development of more effective interventions against neurodegeneration [9–11]. While arterial spin-labeling MR or [^15^O]-water PET are the standard methods for quantifying cerebral perfusion, the early uptake phase for many molecular imaging tracers contains information about the perfusion rate and may thus contribute to the A/T/N schema. Thus, PET imaging with amyloid-β or tau tracers can serve a dual purpose for molecular imaging while also providing a surrogate marker of neurodegeneration. However, no study to date has investigated the comparability of early-phase imaging of tau-PET versus Aβ-PET as surrogates for impaired perfusion and metabolism. We therefore set out to compare the properties of early-phase β-amyloid-PET with [^18^F]flutemetamol versus corresponding findings for tau-PET with the second-generation tracer [^18^F]PI-2620. We tested the hypothesis that the two tracers would be similarly fit for detecting perfusion defects in the early-phase recordings of patients with suspected neurodegenerative disease.

## Methods

### Patient enrolment

All patients who underwent dynamic PET recordings with [^18^F]flutemetamol and [^18^F]PI-2620 for differential diagnosis of AD and other neurodegenerative diseases at the Department of Nuclear Medicine, LMU Munich, seen between October 2018 and August 2021 were included in this study. An orienting sample size estimation (explained σ/residual σ = 1; α = 0.05; G*Power V3.1.9.2) indicated a necessary sample size of *n* = 16 to detect associations between the early-phase of [^18^F]flutemetamol and early-phase of [^18^F]PI-2620 with a statistical power of 0.95. PET imaging was either performed during the clinical workup or part of the screening/imaging of a prospective observational cohort study [12, 13]. Informed written consent was obtained according to the Declaration of Helsinki. The retrospective study was approved by the institutional human experimental ethics committee (application number 17–569).

### Patient collective

Sixty-four patients underwent two-phase [^18^F]flutemetamol-PET and dynamic [^18^F]PI-2620-PET investigations, with an average time gap of 3.2 months (range, 37–545 days) (Table 1). All patients had been referred for PET imaging by local specialists due to suspicion of neurodegenerative disease. The mean age was 69 years (± 8.5; range, 46–87). Twenty-five patients were clinically diagnosed with corticobasal syndrome (CBS) [14], 21 with AD [8], ten with progressive supranuclear palsy (PSP) [15], one with primary progressive aphasia, two with frontotemporal dementia [16, 17], one with Parkinson’s disease, one with vascular dementia, two with subjective memory complaints, and one with mild cognitive impairment without underlying AD pathology. For AD patients, the positive Aβ status was either obtained by PET (15/21 positive) or cerebrospinal fluid (positive for the six remaining patients). For AD patients with no available mini-mental state examination (MMSE) score but recorded Montreal Cognitive Assessment (MoCA) score, MoCA scores were converted into an equivalent MMSE score [18]. Patients with 4R-tauopathies (PSP and CBS) were assessed by the PSP rating scale. Tau-PET analysis and separate analysis of early-phase imaging of both tracers were reported in previous investigations [9, 13, 23–25]. The control cohort (*n* = 15) was selected from previous studies [13, 23]. All controls had negative amyloid- and tau-PET scans (visual read) and a Fazekas score ≤ 1. The mean age of the control cohort was 67.4 ± 9.5 years and none of the controls had memory impairment (MMSE, 29.2 ± 0.8) or motor symptoms.

**Table 1:** Characteristics of the investigated cohort

	AD	4RT	Others	Controls
*n*	21	35	8	15
Subgroups	*n* = 5 SCD *n* = 10 MCI *n* = 6 ADD	10 PSP 25 CBS	2 SCD 1 MCI 2 FTD 1 vascular dementia 1 PPA 1 Parkinson’s disease	n. a
Age (y)	67.7 ± 7.4	69.7 ± 9.5	70.5 ± 6.0	67.4 ± 9.5
Sex	9 female/12 male	21 female/14 male	3 female/5 male	8 female/7 male
PSP rating scale	18.5 ± 2.5 (*n* = 2)	27.5 ± 14.5 (*n* = 31)	25.0 ± 5.0 (*n* = 2)	n. a
MMSE	26.2 ± 4.0 (*n* = 18)	23.0 ± 6.3 (*n* = 23)	28.5 ± 1.5 (*n* = 2)	29.2 ± 0.8 (*n* = 12)
MoCA	22.3 ± 6.4 (*n* = 4)	22.4 ± 6.8 (*n* = 24)	21.0 (*n* = 1)	n. a

*AD*, Alzheimer’s disease; *ADD*, AD dementia; *4RT*, 4-repeat tauopathies; *SCD*, subjective cognitive decline; *MCI*, mild cognitive impairment; *PSP*, progressive supranuclear palsy; *CBS*, corticobasal syndrome; *FTD*, frontotemporal dementia; *PPA*, primary progressive aphasia; *MMSE*, mini-mental state examination; *MoCA*, Montreal Cognitive Assessment

### Radiosynthesis and image acquisition

Radiosynthesis of [^18^F]flutemetamol was performed as described previously [26]. [^18^F]PI-2620 was synthesized using a BOC-protected nitro-precursor automated synthesis module (IBA, Synthera). After semipreparative high-performance liquid chromatography, radiochemical purity was > 97%. Yields were about 30% with a molar activity of 3 × 10^6^ GBq/mmol.

PET data were acquired with a Biograph 64 or a Siemens mCT PET/CT scanner (Siemens Healthineers, Erlangen, Germany) at the Department of Nuclear Medicine, LMU Munich. The [^18^F]flutemetamol acquisition was conducted in two phases, with a perfusion-weighted scan during 0–10 min post-injection, followed by a second dynamic recording during 90–110 min post-injection [27]. The [^18^F]PI-2620-PET acquisition consisted of a dynamic recording during 0–60 min post-injection. The mean injected activity was 191 MBq (range, 151–223 MBq) for [^18^F]PI-2620-PET and 185 MBq (range, 124–219 MBq) for [^18^F]flutemetamol.

### Image processing

Perfusion phase images were constructed during 0–10 min p.i. for [^18^F]flutemetamol-PET and 0.5–2.5 min p.i. for [^18^F]PI-2620-PET and summarized to a single frame, following precedent for the selection of the perfusion-weighted evaluations [9, 27]. Here, robust correlations between FDG-PET and different early time windows of amyloid-PET were observed, whereas the correlation between FDG-PET and early phase [^18^F]PI-2620-PET was optimal for very early frames but decreased distinctly for acquisition times > 5 min p.i. For spatial normalization, perfusion-weighted images were registered to the template of a healthy control cohort using the PMOD Fusion tool (version 3.5, PMOD Technologies, Zurich, Switzerland). For each scan, we obtained segmentation of the SUV maps for each of the 246 brain regions defined by the Brainnetome Atlas [28], which were extracted using the PMOD View tool. For visualization of the local perfusion deficit, three-dimensional stereotactic surface projections (3D-SSP) [29] were created using the software Neurostat (Department of Radiology, University of Washington, Seattle, WA, USA). 3D-SSP visualizations were not analyzed but used for illustration purposes.

### Statistical analysis

SUV ratios (SUVr) were computed relative to the respective global mean uptake and cerebellar uptake using a MATLAB (version R2016a, The MathWorks, Portola Valley, CA, USA) script [9, 27, 30]. Regional group differences in tracer uptake (z-scores) were computed for both tracers by contrasting the mean SUVrs by region in the patient versus control groups using MATLAB as well. Pearson’s correlation coefficients between respective z-scores of early-phase [^18^F]flutemetamol- and [^18^F]PI-2620-PET recordings were evaluated by region and lobes with GraphPad Prism (version 8.4.3 (686)) after confirming normal distribution by a Kolmogorov–Smirnov test. We also evaluated the mean lobular z-scores in bilateral frontal (*n* = 34 Brainnetome regions each side), temporal (*n* = 28 regions each side), parietal (*n* = 19 regions for each side), and occipital regions (*n* = 11 regions each side). Patients with significant neuronal injury (> 5 regions with z-scores <  − 3) and patients with different entities of neurodegenerative diseases (AD vs. 4R-tauopathies) served for subgroup analysis. For evaluation of the dependence of the correlation between early phases of tau-PET and amyloid-PET of the regional neuronal injury burden and the volumes of the region, Fisher’s transformation was used. Partial regression corrected for age and gender was executed to evaluate the relation between symptom severity and perfusion deficit using MATLAB. A *X*^2^ test was applied to test for similarity of [^18^F]PI-2620 and [^18^F]flutemetamol regions with a significant perfusion surrogate correlation with clinical severity.

## Results

### Regional comparison of early-phase [^18^F]flutemetamol and early-phase [^18^F]PI-2620 in different brain regions

**Fig. 1:**
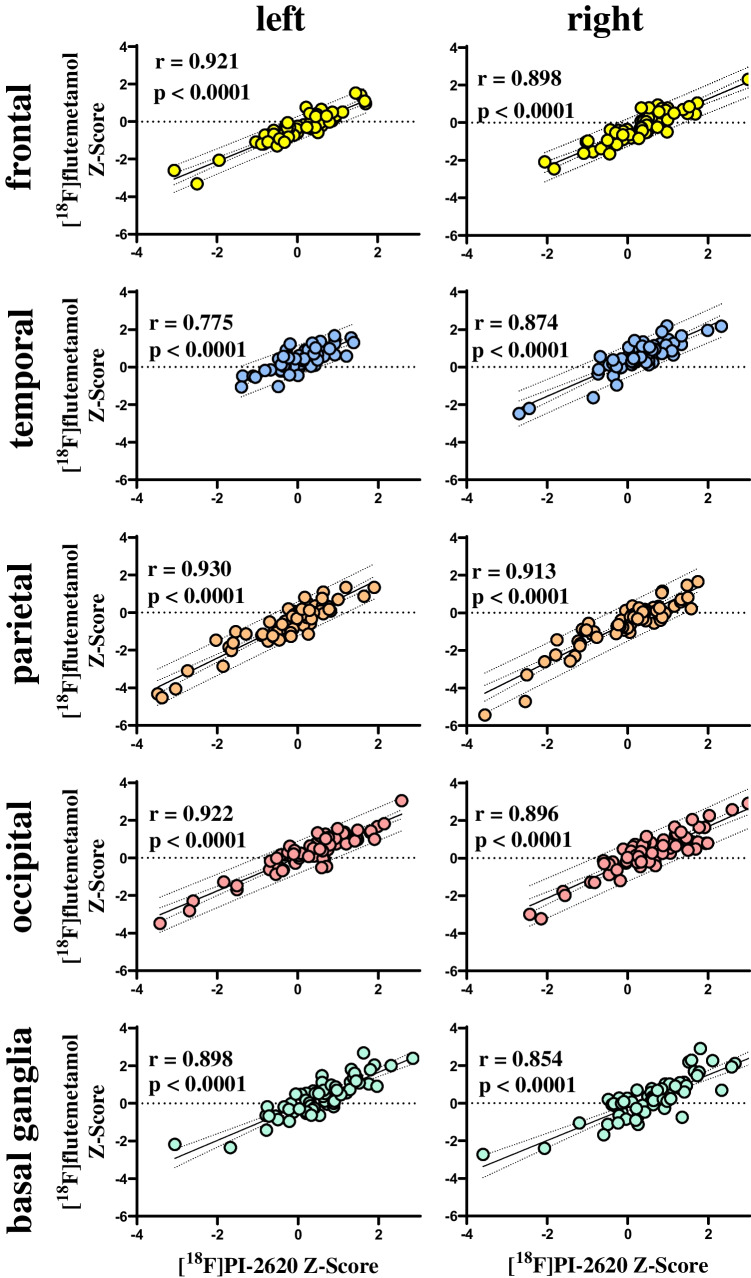
Correlation of early-phase [^18^F]flutemetamol and [^18^F]PI-2620 uptake in different brain regions of patients with neurodegenerative diseases after normalization to global mean SUV and z-score computation against a group of healthy controls. Left column, left brain; right column, right brain; the rows show the frontal, temporal, parietal, and occipital brain region as well as the basal ganglia from the top down

All cortical brain regions and the evaluated subcortical regions (basal ganglia) showed high correlations of early-phase [^18^F]PI-2620 and [^18^F]flutemetamol SUVr values, with the highest correlations in the left parietal lobe (*r* = 0.930; *p* < 0.0001) and the lowest in the left temporal lobe (*r* = 0.775; *p* < 0.0001). Correlation plots per lobe are shown in Fig. 1. There was no significant difference between single region *r* coefficients for global mean (*r* = 0.819 ± 0.316) and cerebellar scaling (*r* = 0.820 ± 0.262, *p* = 0.532). The correlations for all single regions using global mean scaling as well as cerebellar scaling (as a cross-validation) are shown in Supplementary Tables 1 and 2.

### Correlation between early phases of tau-PET and amyloid-PET in dependence of the regional neuronal injury burden

**Fig. 2:**
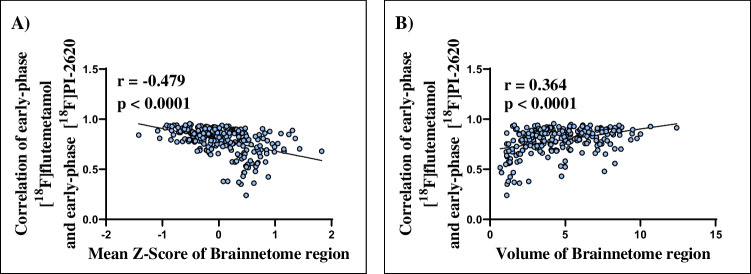
**A** Regions with a less severe perfusion deficit as indicated by a higher z-score show lower correlation between the early-phase [^18^F]flutemetamol and [^18^F]PI-2620-PET readouts than in regions with more severe perfusion deficit. **B** Larger regions show a higher correlation of early-phase [^18^F]flutemetamol and [^18^F]PI-2620-PET readouts as compared to smaller regions

The agreement between early-phase tau-PET and amyloid-PET results was significantly lower in regions with less severe perfusion defects as indicated by lower z-score deviation: there was a moderate correlation of the mean z-score of the individual Brainnetome regions (*n* = 246) assessed in [^18^F]flutemetamol-PET and [^18^F]PI-2620-PET and the correlation of both early-phase indices in the specific Brainnetome regions (*r* = 0.479; *p* < 0.0001). Figure 2A shows the mean regional z-scores against the regional correlations for both early-phase PET recordings.

### Correlation between early phases of tau-PET and amyloid-PET in dependence of the region volume

The predefined Brainnetome regions had volumes between 0.67 and 12.41 cm^3^. Correlations between early phase uptakes of the two tracers were higher in the larger brain regions the (Fig. 2B), giving a moderate positive correlation between the correlation between early-phase [^18^F]flutemetamol-PET and [^18^F]PI-2620-PET z-scores as a function of the size of region when evaluating global mean scaled data (*r* = 0.364; *p* < 0.0001).

### Correlation of early phases of tau-PET and amyloid-PET in specific subgroups of patients with Alzheimer’s disease and 4-repeat tauopathies

**Fig. 3:**
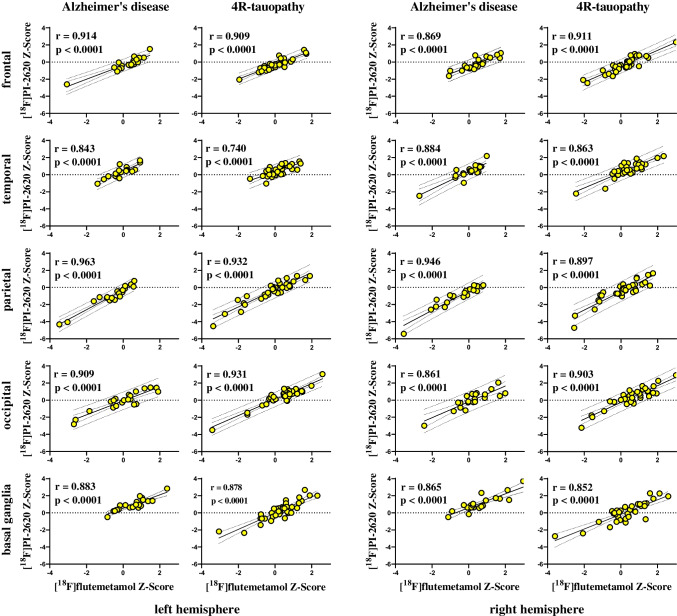
Correlation of early-phase [^18^F]flutemetamol and [^18^F]PI-2620 z-scores in different brain regions for subgroups of patients with Alzheimer’s disease and patients with 4R-tauopathies. Left two columns, left cerebral hemisphere; right two columns, right cerebral hemisphere; first and third column, patients with Alzheimer’s disease; second and fourth column, patients with 4R-tauopathies; the rows show the frontal, temporal, parietal, and occipital brain region as well as the basal ganglia, proceeding from the top down

The correlation of perfusion surrogates using early-phase [^18^F]flutemetamol-PET [^18^F]PI-2620-PET did not significantly differ (*p* = 0.627) between patients with AD (*r* = 0.843–0.963) and patients with 4-repeat tauopathies (*r* = 0.740–0.932) (Fig. 3). In particular, patients with AD showed the highest correlations in the parietal lobe, the frontal lobe (excluding the motor strip), the anterior cingulate gyrus, the putamen, and the caudate nucleus, as well as the superior and middle temporal gyrus, equally for both tracers. Patients with 4-repeat tauopathies showed the highest correlation in the parietal lobe, the frontal lobe, the anterior and the posterior cingulate gyrus, and the caudate nucleus and the putamen, as well as the insula and the mesial occipital lobe, irrespective of the used tracer. Evaluation of the remaining eight patients without a clinical diagnosis of AD or 4-repeat tauopathy also showed a high correlation in all evaluated subregions (*r* = 0.778–0.979).

### Correlation between cognitive screening performance and neuronal injury assessment by early-phase tau- and amyloid-PET in patients with Alzheimer’s disease

**Fig. 4:**
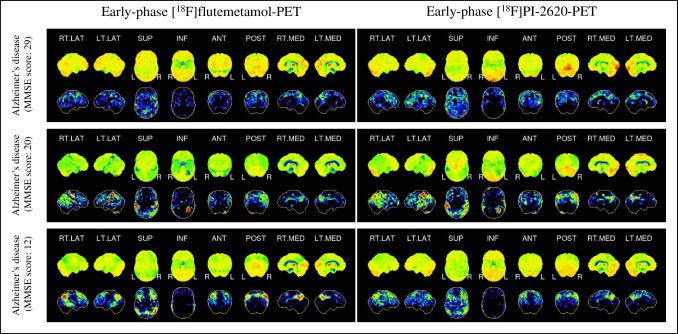
Visual comparison of early-phase [^18^F]flutemetamol (left side) and early-phase [.^18^F]PI-2620 (right side) PET images (three-dimensional stereotactic surface projections) in patients with Alzheimer’s disease. Upper row, patient with minor symptoms (MMSE score, 29). Middle row, patient with moderate symptoms (MMSE score, 20); lower row, patient with severe symptoms (MMSE score, 12)

For 20 patients diagnosed with AD, MMSE or converted MoCA scores were available at the time of imaging (Table 1). Partial regression corrected for age and gender showed that cognitive assessment was a significant predictor (*p* < 0.05) for the early-phase [^18^F]flutemetamol z-score results in a total of 67/246 Brainnetome regions, especially in the parietal cortex, the precuneus, and the posterior cingulate gyrus (21/38 regions) (Fig. 4). The single region partial correlation coefficient rho ranged from 0.473 to 0.851 (median = 0.581). The partial regression evaluated for the perfusion phase of [^18^F]PI-2620 PET showed a significant correlation of cognitive assessment and the z-scores in the same lobes in a total of 66/246 Brainnetome regions), e.g., in the parietal cortex, the precuneus, and the posterior cingulate gyrus (25/38 regions). The absolute magnitude of rho ranged from 0.470 to 0.814 (median = 0.579). The regions where z-scores correlated significantly with cognitive assessment were highly similar for early phase tau- and β-amyloid-PET (*X*^2^ = 0.01).

### Correlation between symptom severity and neuronal injury assessment by early-phase tau- and amyloid-PET in patients with 4-repeat tauopathies

**Fig. 5:**
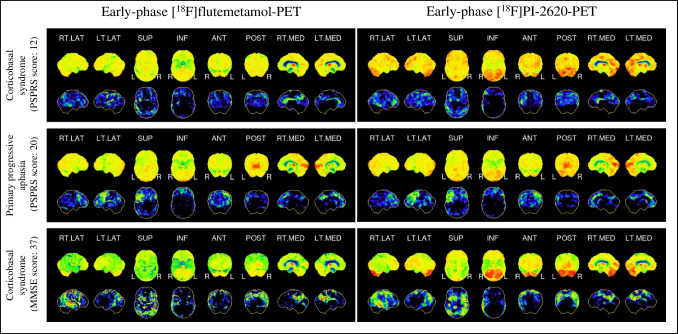
Visual comparison of early-phase [^18^F]flutemetamol (left side) and early-phase [^18^F]PI-2620 (right side) PET images (three-dimensional stereotactic surface projections) in patients with 4R-tauopathies. Upper row, patient with corticobasal syndrome with minor symptoms and low PSPRS score (12). Middle row, patient with primary progressive aphasia with moderately severe symptoms (PSPRS score, 20); lower row, patient with corticobasal syndrome with severe symptoms (PSPRS score, 37). All patients indicated 4R-like tau positivity in the late-phase [^18^F]PI-2620 tau-PET

Thirty-one patients with 4-repeat tauopathies had been assessed according to the PSP rating scale (PSPRS) at the time of imaging (Fig. 5). PSPRS scores were a significant predictor (*p* < 0.05) of the apparent perfusion deficit of 21/246 regions in [^18^F]flutemetamol- and 19/246 regions in [^18^F]PI-2620-PET. The affected regions in these patients consisted mainly of subcortical regions, including the caudate nucleus and the thalamus (4/28 in [^18^F]flutemetamol-PET and 7/28 in [^18^F]PI-2620-PET), as well as the anterior cingulate gyrus (3/8 in [^18^F]flutemetamol-PET and 5/8 in [^18^F]PI-2620-PET). Additionally, a few regions of the (inferior) frontal lobe showed a significant correlation (10/68 in [^18^F]flutemetamol-PET and 5/68 in [^18^F]PI-2620-PET). The rho of single regions ranged between 0.39 and -0.65 (median, -0.43). The regions where z-scores correlated significantly with PSPRS scores were similar for early-phase tau- and amyloid-PET (*X*^2^ = 0.11).

## Discussion

Previous studies have shown a high correlation between the perfusion phase uptake of β-amyloid and tau-PET tracers with the extent of regional neuronal injury to FDG-PET [9–11, 24, 31–33]. These relationships are relevant in the context of flow–metabolism coupling and the cerebrometabolic deficits occurring in AD and other neurodegenerative conditions. Indeed, cerebral perfusion-PET still has a role in the diagnosis of AD [34], although the regional relationship between perfusion and metabolic rate is imperfect [35]. A comprehensive imaging biomarker-based diagnostic workup in patients with neurodegenerative diseases may include FDG-PET, as well as β-amyloid-PET and tau-PET, for a classification within the A/T/N scheme, namely, amyloid-PET or CSF β-amyloid (A), CSF p-tau (T), and MRI-based medial temporal lobe atrophy (N) [7]. The opportunity for “one-stop-shop” PET examinations providing information about specific pathology and general decline in cerebral perfusion/metabolism within the setting of a single PET examination would offer many advantages both for the patient and for healthcare systems: first, a time-consuming and costly examination can be spared. Second, the radiation exposure to patients is reduced. Finally, there is no requirement for fasting prior to β-amyloid or tau-PET, which could be problematic for patients with poor glucose control. The current results additionally suggest the possibility of using early-phase β-amyloid-PET or early-phase tau-PET as surrogates for perfusion imaging. Comparable assessment of regional neuronal injury by early phase of [^18^F]PI-2620-tau- and [^18^F]flutemetamol-amyloid-PET potentially enable tracking longitudinal changes by serial PET examination with different tracers at different time points. This has clinical relevance since β-amyloid-PET can serve as early diagnostic biomarker of AD pathology [36]. On the other hand, tau-PET is more likely to be employed in patients with more advanced cognitive decline, since tau-PET signals have a more robust correlation with cognitive impairment [37].

For evaluation of the comparability of early-phase [^18^F]flutemetamol-PET and [^18^F]PI-2620-PET as markers of perfusion deficit, we investigated the correlation of the two markers across single brain regions. For subgroup analysis, we divided the patients into groups with AD or 4-repeat tauopathies. Furthermore, we evaluated the impact on these relationships of volume of evaluated regions, severity of perfusion deficit, and clinical symptoms using established clinical scores. We found strong to excellent correlations between early phases of [^18^F]PI-2620-tau-PET and [^18^F]flutemetamol-amyloid-PET in the neocortical regions and in most of subcortical regions. In general, the correlation was the strongest in lobes, regions, and patients with a significant perfusion deficit. First, there was a moderate negative correlation of the z-score in single Brainnetome regions and the correlations between early-phase β-amyloid-PET and tau-PET, thus showing that a more severe perfusion deficit was associated with a higher correlation between early-phase [^18^F]PI-2620-PET and [^18^F]flutemetamol-PET. Second, among the four great lobes, the correlations between earl- phase β-amyloid-PET and tau-PET were the highest in the frontal and parietal lobes. This is in line with more severe neuronal damage in the frontal lobe, manifesting in a lower average z-score (frontal, -0.315; parietal, -0.615; temporal, +0.485; occipital, +0.310). Third, sub-analysis of single Brainnetome regions showed the best correlation in known predilection sites of neuronal damage in patients with Alzheimer’s disease, e.g., the precuneus [38]. One reason for the improved comparability of the perfusion deficit assessment of both tracers in regions with a more severe perfusion deficit might result from an increased perfusion deficit-to-noise ratio. Higher perfusion deficit-to-noise ratios counteract the variance of PET imaging results, which is driven by confounding physical effects like attenuation, scattering, or random-coincidence events [39, 40]. We note that the perfusion phase of [^18^F]flutemetamol-PET is less subject to noise when compared to the perfusion phase of [^18^F]PI-2620-PET, due to the longer time frame of assessment. Thus, early-phase [^18^F]flutemetamol-PET may have advantage to detect smaller perfusion deficits when compared to early-phase [^18^F]PI-2620-PET.

In another sub-analysis, the perfusion surrogates from the two tracers correlated equally well in patients with different entities of neurodegenerative diseases, e.g., AD and 4-repeat tauopathies, which shows the robustness of our approach irrespective of the particular diagnosis. This result is consistent with recent findings showing that FDG-PET and the perfusion phases of β-amyloid or tau-PET were comparable for identifying patients with different neurodegenerative diseases [9, 11, 27].

Additionally, we investigated the correlation between perfusion surrogates as a function of the volume of the brain region. The two perfusion surrogates were more similar in larger regions than in small regions. That relationship is explainable by the greater quantitative robustness and lesser vulnerability to partial volume effects of larger structures.

Furthermore, we investigated the partial regression of clinical severity scores for AD and PSP with the z-scores in the Brainnetome regions, corrected for age and gender. We found that in the AD group, the MMSE scores were a significant predictor for z-scores in AD predilection sites. Patients with 4-repeat tauopathies similarly showed a significant relationship between their PSPRS scores and the corresponding z-scores in PSP signature regions like the basal ganglia and the limbic lobe. This is in accord with recent results from our group indicating a high correlation between perfusion deficit and the PSP rating scale scores in patients with 4R-tauopathies [25]. Thus, perfusion-weighted imaging with [^18^F]flutemetamol or [^18^F]PI-2620 can serve for monitoring disease severity, irrespective of their properties as molecular imaging agents with specific for amyloid-β or tau. The slightly ameliorated correlation of clinical performance and [^18^F]flutemetamol-amyloid-PET compared to [^18^F]PI-2620-tau-PET might be explainable with a shorter interval between the clinical assessment and acquisition of [^18^F]flutemetamol-amyloid-PET (90 ± 98 days vs. 181 ± 187 days).

The potential limitations of this study arise from the variable time interval between acquisitions of the early-phase β-amyloid and tau-PET imaging. A gap of as much as 18 months might well have been accompanied by disease progression, to the detriment of correlations between early phase PET results. Another limiting factor in this study is the lack of histopathological data, which might have established a certain diagnosis. Furthermore, we only considered cognitive screening at the time of imaging which resulted in some missing MMSE scores in the AD cohort. However, given the known strong association between neuronal injury and cognitive performance in AD [41, 42], this sample size was sufficient to assess the comparability of regional early-phase tau-PET and early-phase amyloid-PET correlations with MMSE.

Our use of early tracer uptake as a surrogate for cerebral perfusion calls for some consideration. The initial brain uptake of any PET tracer is a function of the tracer’s permeability surface area product and the extraction fraction, which is the ratio of the unidirectional blood brain clearance (K1) to the perfusion rate, F. As such, recordings of initial tracer uptake are an imperfect surrogate of the rate of cerebral blood flow, which is best depicted by tracers with a high extraction fraction. For example, we have shown that initial brain uptake of the dopamine receptor ligand [^18^F]fallypride correlated with a more direct index of perfusion rate, thus presenting a useful surrogate for age-dependent reductions in perfusion [43]. Additionally, there was a good correlation between the early perfusion phase uptake of the monoamine oxidase B ligand [^11^C]deprenyl-d_1_ with that of the β-amyloid tracer [^11^C]PiB in the same individuals PET [19, 20]. Thus, the high initial uptake of [^18^F]flutemetamol in rodent brain, peaking at SUV = 5.8 at 2 min post-injection [21], and similarly high uptake for [^18^F]PI-2620 in non-human primate brain [22] are indicative of high extraction fraction, which is a necessary precondition for using early-phase uptake as a surrogate of perfusion rate. Furthermore, we note that reduced perfusion can also represent remote effects without local neuronal injuries, which needs to be considered in the interpretation of perfusion surrogates as “N” biomarkers.

## Conclusions

The early phases of [^18^F]PI-2620-tau-PET and [^18^F]flutemetamol-amyloid-PET brain uptake are comparable surrogates of cerebral perfusion deficit, as a biomarker for neuronal injury in neurodegenerative disorders. Uptake in regions of a highly parcellated brain atlas indicated robust agreement between early phases of tau-PET and amyloid-PET, especially in signature regions of significant neuronal damage in AD and PSP.

## Supplementary Information

Below is the link to the electronic supplementary material.

Supplementary file1 (DOCX 33 KB)

## Data Availability

The raw data supporting the conclusions of this article will be made available by the authors upon request to the corresponding author.
