# Supplementary material for: Assessment of perfusion deficit with early phases of [18F]PI-2620 tau-PET versus [18F]flutemetamol-amyloid-PET recordings
Source: Eur J Nucl Med Mol Imaging. 2022 Dec 27;50(5):1384–94. doi: 10.1007/s00259-022-06087-y (PMC10027797; doi:10.1007/s00259-022-06087-y)
Supplement: Supplementary file 1 — Supplementary file1 (DOCX 33 KB) [file 259_2022_6087_MOESM1_ESM.docx]

**Supplementary table 1.** Correlation of mean Z-Scores of 64 patients of early-phase [^18^F]PI-2620 and early-phase [^18^F]flutemetamol using global mean scaling.

| Brainnetome region | mean Z-Score PI-2620 | mean Z-Score flutemetamol | Pearson's correlation coefficient |
| --- | --- | --- | --- |
| 1 | -0.743 | -0.960 | 0.941 |
| 2 | -0.950 | -0.908 | 0.914 |
| 3 | -0.537 | -0.321 | 0.904 |
| 4 | -0.494 | -0.272 | 0.858 |
| 5 | -0.312 | 0.670 | 0.848 |
| 6 | -0.343 | 0.366 | 0.784 |
| 7 | -0.334 | 0.007 | 0.706 |
| 8 | -0.641 | -0.373 | 0.778 |
| 9 | -0.647 | -0.708 | 0.911 |
| 10 | -0.467 | -0.366 | 0.814 |
| 11 | -0.491 | -0.759 | 0.947 |
| 12 | -0.337 | -0.514 | 0.893 |
| 13 | 0.178 | 0.157 | 0.929 |
| 14 | 0.257 | 0.245 | 0.899 |
| 15 | -0.069 | 0.284 | 0.920 |
| 16 | -0.408 | -0.054 | 0.845 |
| 17 | -0.612 | -0.266 | 0.913 |
| 18 | -1.084 | -0.673 | 0.884 |
| 19 | 0.278 | 0.904 | 0.896 |
| 20 | 0.331 | 1.031 | 0.860 |
| 21 | -0.093 | 0.321 | 0.924 |
| 22 | 0.077 | 0.827 | 0.802 |
| 23 | -0.579 | -0.264 | 0.903 |
| 24 | -0.296 | 0.437 | 0.777 |
| 25 | -0.740 | -0.278 | 0.774 |
| 26 | -0.711 | -0.383 | 0.870 |
| 27 | 0.447 | 0.994 | 0.892 |
| 28 | 0.469 | 1.087 | 0.909 |
| 29 | -0.166 | 0.033 | 0.842 |
| 30 | -0.266 | 0.075 | 0.831 |
| 31 | -0.097 | 0.372 | 0.855 |
| 32 | 0.050 | 0.516 | 0.790 |
| 33 | -0.370 | 0.148 | 0.821 |
| 34 | -0.083 | 0.684 | 0.826 |
| 35 | -0.315 | 0.408 | 0.883 |
| 36 | -0.046 | 0.805 | 0.872 |
| 37 | -0.190 | -0.176 | 0.910 |
| 38 | 0.390 | 0.198 | 0.899 |
| 39 | -0.274 | -0.382 | 0.850 |
| 40 | -0.129 | 0.196 | 0.812 |
| 41 | 0.298 | 0.185 | 0.900 |
| 42 | 0.716 | 0.631 | 0.845 |
| 43 | 0.031 | -0.111 | 0.825 |
| 44 | 0.363 | 0.565 | 0.780 |
| 45 | 0.860 | 1.041 | 0.786 |
| 46 | 1.357 | 1.470 | 0.808 |
| 47 | 0.788 | 0.836 | 0.808 |
| 48 | 0.907 | 0.938 | 0.878 |
| 49 | 0.952 | 0.119 | 0.754 |
| 50 | 1.047 | 0.112 | 0.756 |
| 51 | -0.177 | -0.032 | 0.894 |
| 52 | 0.208 | 0.352 | 0.878 |
| 53 | -0.051 | 0.529 | 0.735 |
| 54 | 0.222 | 1.237 | 0.692 |
| 55 | -0.602 | -0.326 | 0.803 |
| 56 | -0.258 | -0.071 | 0.818 |
| 57 | 0.181 | 0.413 | 0.832 |
| 58 | -0.265 | 0.086 | 0.791 |
| 59 | -0.219 | 0.090 | 0.691 |
| 60 | -0.169 | 0.133 | 0.663 |
| 61 | 0.044 | -0.189 | 0.866 |
| 62 | 0.324 | 0.268 | 0.872 |
| 63 | -0.543 | -0.099 | 0.795 |
| 64 | -0.214 | 0.314 | 0.691 |
| 65 | -0.314 | -0.463 | 0.935 |
| 66 | -0.317 | -0.375 | 0.867 |
| 67 | -0.373 | -0.593 | 0.921 |
| 68 | -0.166 | -0.190 | 0.876 |
| 69 | 0.403 | -0.158 | 0.787 |
| 70 | 0.748 | -0.040 | 0.694 |
| 71 | -0.154 | -0.145 | 0.876 |
| 72 | 0.201 | 0.182 | 0.864 |
| 73 | -0.148 | -0.633 | 0.911 |
| 74 | -0.225 | -0.788 | 0.927 |
| 75 | -0.106 | 0.396 | 0.747 |
| 76 | 0.287 | 1.454 | 0.773 |
| 77 | -0.029 | -0.716 | 0.751 |
| 78 | 0.129 | -0.653 | 0.909 |
| 79 | 0.368 | 0.489 | 0.765 |
| 80 | 0.129 | 0.012 | 0.776 |
| 81 | -0.533 | 0.080 | 0.778 |
| 82 | -0.045 | 0.749 | 0.787 |
| 83 | 0.246 | 0.764 | 0.759 |
| 84 | 0.367 | 0.905 | 0.850 |
| 85 | -1.048 | -0.130 | 0.809 |
| 86 | -0.121 | 0.748 | 0.807 |
| 87 | -0.181 | -0.060 | 0.855 |
| 88 | -0.064 | -0.060 | 0.856 |
| 89 | 0.990 | 0.911 | 0.724 |
| 90 | 0.625 | 0.635 | 0.675 |
| 91 | 0.081 | -0.378 | 0.379 |
| 92 | 0.623 | 0.102 | 0.361 |
| 93 | 0.848 | 0.947 | 0.694 |
| 94 | 1.826 | 1.800 | 0.680 |
| 95 | 0.257 | 0.528 | 0.756 |
| 96 | 0.494 | 0.886 | 0.790 |
| 97 | -0.319 | -0.111 | 0.806 |
| 98 | 0.169 | 0.551 | 0.762 |
| 99 | -0.181 | 0.025 | 0.682 |
| 100 | 0.207 | 0.784 | 0.715 |
| 101 | 0.697 | 0.712 | 0.688 |
| 102 | 1.159 | 1.307 | 0.682 |
| 103 | 1.435 | 0.501 | 0.704 |
| 104 | 1.154 | 0.294 | 0.702 |
| 105 | 0.491 | -0.038 | 0.691 |
| 106 | 0.448 | 0.059 | 0.665 |
| 107 | 0.463 | 0.027 | 0.652 |
| 108 | 0.721 | 0.255 | 0.661 |
| 109 | 0.409 | -0.017 | 0.606 |
| 110 | 0.988 | 0.546 | 0.674 |
| 111 | 0.587 | -0.083 | 0.559 |
| 112 | 0.695 | 0.050 | 0.567 |
| 113 | 0.878 | 0.138 | 0.424 |
| 114 | 0.740 | 0.326 | 0.626 |
| 115 | 0.448 | -0.512 | 0.469 |
| 116 | 0.486 | -0.304 | 0.241 |
| 117 | 0.457 | -0.340 | 0.467 |
| 118 | 0.394 | -0.423 | 0.351 |
| 119 | 0.243 | -0.476 | 0.753 |
| 120 | 0.238 | -0.528 | 0.575 |
| 121 | -0.087 | -0.191 | 0.869 |
| 122 | -0.313 | -0.706 | 0.853 |
| 123 | -0.042 | -0.070 | 0.832 |
| 124 | -0.457 | -0.605 | 0.787 |
| 125 | -0.469 | -0.189 | 0.856 |
| 126 | -0.683 | -0.219 | 0.867 |
| 127 | -0.563 | -0.299 | 0.887 |
| 128 | -0.042 | 0.312 | 0.917 |
| 129 | -0.587 | -0.506 | 0.917 |
| 130 | -0.798 | -0.816 | 0.939 |
| 131 | -0.058 | 0.061 | 0.889 |
| 132 | -0.465 | -0.330 | 0.857 |
| 133 | -0.207 | -0.112 | 0.908 |
| 134 | -0.066 | 0.014 | 0.922 |
| 135 | -0.174 | 0.253 | 0.915 |
| 136 | -0.322 | 0.333 | 0.896 |
| 137 | -0.920 | -0.633 | 0.939 |
| 138 | -0.497 | -0.094 | 0.886 |
| 139 | -0.836 | -0.707 | 0.887 |
| 140 | -0.582 | -0.488 | 0.879 |
| 141 | -1.418 | -0.578 | 0.842 |
| 142 | -0.848 | 0.427 | 0.794 |
| 143 | -0.514 | -0.225 | 0.915 |
| 144 | -0.548 | -0.269 | 0.927 |
| 145 | -0.364 | -0.220 | 0.885 |
| 146 | -0.309 | -0.218 | 0.852 |
| 147 | -0.583 | -0.798 | 0.916 |
| 148 | -0.155 | -0.250 | 0.905 |
| 149 | -0.200 | -0.306 | 0.924 |
| 150 | -0.351 | -0.458 | 0.931 |
| 151 | 0.004 | -0.098 | 0.910 |
| 152 | 0.056 | 0.250 | 0.938 |
| 153 | -0.681 | -0.931 | 0.880 |
| 154 | -0.461 | -0.616 | 0.871 |
| 155 | -0.427 | 0.251 | 0.773 |
| 156 | -0.151 | 0.667 | 0.750 |
| 157 | -0.034 | -0.219 | 0.829 |
| 158 | 0.475 | 0.177 | 0.853 |
| 159 | -0.616 | -0.424 | 0.894 |
| 160 | -0.504 | -0.219 | 0.797 |
| 161 | -0.271 | 0.001 | 0.859 |
| 162 | -0.117 | 0.258 | 0.795 |
| 163 | 0.126 | -0.030 | 0.737 |
| 164 | -0.024 | -0.320 | 0.864 |
| 165 | -0.144 | -0.712 | 0.647 |
| 166 | 0.295 | -0.319 | 0.573 |
| 167 | -0.085 | -0.240 | 0.785 |
| 168 | 0.398 | 0.090 | 0.856 |
| 169 | -0.334 | -1.048 | 0.689 |
| 170 | 0.205 | -0.344 | 0.770 |
| 171 | 0.454 | -0.004 | 0.796 |
| 172 | 0.396 | 0.116 | 0.773 |
| 173 | -0.187 | -0.657 | 0.723 |
| 174 | 0.695 | 0.177 | 0.797 |
| 175 | -0.415 | -0.500 | 0.782 |
| 176 | -0.549 | -0.633 | 0.895 |
| 177 | -0.866 | -1.041 | 0.932 |
| 178 | -0.721 | -1.103 | 0.916 |
| 179 | -0.607 | -0.817 | 0.902 |
| 180 | -0.337 | -0.398 | 0.838 |
| 181 | 0.160 | -0.389 | 0.793 |
| 182 | -0.278 | -0.603 | 0.813 |
| 183 | -0.803 | -0.837 | 0.844 |
| 184 | -0.520 | -0.373 | 0.795 |
| 185 | -0.422 | -0.522 | 0.859 |
| 186 | -0.436 | -0.359 | 0.855 |
| 187 | 0.354 | -0.314 | 0.824 |
| 188 | 0.008 | -0.263 | 0.818 |
| 189 | 0.573 | -0.010 | 0.746 |
| 190 | 0.658 | 0.068 | 0.528 |
| 191 | 0.490 | 0.184 | 0.829 |
| 192 | 0.650 | 0.403 | 0.829 |
| 193 | 0.316 | 0.008 | 0.581 |
| 194 | 0.677 | 0.468 | 0.719 |
| 195 | 0.415 | -0.055 | 0.862 |
| 196 | 0.443 | 0.059 | 0.789 |
| 197 | 0.320 | 0.139 | 0.899 |
| 198 | 0.150 | 0.096 | 0.878 |
| 199 | 0.158 | 0.595 | 0.913 |
| 200 | 0.276 | 0.849 | 0.857 |
| 201 | -0.163 | 0.341 | 0.905 |
| 202 | 0.349 | 1.153 | 0.871 |
| 203 | 0.043 | 0.968 | 0.754 |
| 204 | 0.362 | 1.294 | 0.752 |
| 205 | 0.685 | 0.526 | 0.558 |
| 206 | 0.650 | 0.313 | 0.479 |
| 207 | 0.777 | 0.777 | 0.904 |
| 208 | 0.351 | 0.937 | 0.884 |
| 209 | -0.108 | 0.062 | 0.882 |
| 210 | 0.139 | 0.425 | 0.929 |
| 211 | 0.159 | -0.809 | 0.380 |
| 212 | 0.357 | -0.747 | 0.371 |
| 213 | 0.523 | 0.114 | 0.517 |
| 214 | 0.511 | -0.061 | 0.550 |
| 215 | 0.482 | -0.712 | 0.532 |
| 216 | 0.449 | -0.639 | 0.619 |
| 217 | 0.431 | -1.195 | 0.431 |
| 218 | 0.491 | -0.748 | 0.523 |
| 219 | 0.012 | -0.035 | 0.913 |
| 220 | 0.532 | 0.954 | 0.796 |
| 221 | 0.133 | 0.392 | 0.827 |
| 222 | 0.090 | 0.257 | 0.858 |
| 223 | 0.850 | 0.235 | 0.658 |
| 224 | 0.732 | -0.087 | 0.734 |
| 225 | 1.307 | 1.811 | 0.889 |
| 226 | 1.243 | 1.926 | 0.849 |
| 227 | -0.515 | -0.702 | 0.955 |
| 228 | -0.828 | -1.078 | 0.929 |
| 229 | 0.819 | 1.515 | 0.861 |
| 230 | 1.104 | 1.848 | 0.857 |
| 231 | -0.068 | -0.238 | 0.899 |
| 232 | -0.130 | 0.158 | 0.701 |
| 233 | 0.191 | 0.565 | 0.862 |
| 234 | 0.353 | 1.221 | 0.640 |
| 235 | 0.371 | 1.055 | 0.637 |
| 236 | 0.831 | 1.746 | 0.573 |
| 237 | -0.241 | -0.915 | 0.906 |
| 238 | -0.591 | -1.215 | 0.941 |
| 239 | 0.560 | 1.194 | 0.584 |
| 240 | -0.011 | -0.172 | 0.657 |
| 241 | 0.073 | -0.519 | 0.729 |
| 242 | -0.382 | -1.143 | 0.681 |
| 243 | -0.137 | -0.601 | 0.787 |
| 244 | -0.263 | -0.659 | 0.753 |
| 245 | 0.433 | 1.373 | 0.733 |
| 246 | 0.333 | 1.193 | 0.652 |

**Supplementary table 2.** Correlation of mean Z-Scores of 64 patients of early-phase [^18^F]PI-2620 and early-phase [^18^F]flutemetamol using cerebellar mean scaling.

| Brainnetome region | mean Z-Score PI-2620 | mean Z-Score flutemetamol | Pearson's correlation coefficient |
| --- | --- | --- | --- |
| 1 | -1.090 | -0.909 | 0.926 |
| 2 | -1.255 | -0.578 | 0.872 |
| 3 | -1.146 | -0.245 | 0.874 |
| 4 | -1.042 | -0.240 | 0.818 |
| 5 | -1.251 | 0.195 | 0.819 |
| 6 | -1.170 | 0.087 | 0.745 |
| 7 | -1.102 | -0.074 | 0.751 |
| 8 | -1.131 | -0.297 | 0.787 |
| 9 | -1.055 | -0.529 | 0.878 |
| 10 | -0.962 | -0.282 | 0.795 |
| 11 | -0.867 | -0.727 | 0.931 |
| 12 | -0.784 | -0.429 | 0.873 |
| 13 | -0.630 | 0.019 | 0.892 |
| 14 | -0.642 | 0.046 | 0.828 |
| 15 | -0.960 | 0.069 | 0.866 |
| 16 | -1.097 | -0.114 | 0.813 |
| 17 | -1.231 | -0.240 | 0.894 |
| 18 | -1.406 | -0.357 | 0.877 |
| 19 | -0.870 | 0.352 | 0.860 |
| 20 | -1.034 | 0.364 | 0.829 |
| 21 | -0.908 | 0.083 | 0.878 |
| 22 | -1.049 | 0.277 | 0.757 |
| 23 | -1.188 | -0.229 | 0.875 |
| 24 | -1.168 | 0.130 | 0.802 |
| 25 | -1.310 | -0.214 | 0.748 |
| 26 | -1.267 | -0.294 | 0.836 |
| 27 | -0.731 | 0.523 | 0.834 |
| 28 | -0.776 | 0.522 | 0.856 |
| 29 | -1.021 | -0.041 | 0.835 |
| 30 | -1.115 | -0.058 | 0.809 |
| 31 | -1.017 | 0.120 | 0.829 |
| 32 | -0.908 | 0.172 | 0.772 |
| 33 | -1.229 | 0.002 | 0.799 |
| 34 | -1.530 | 0.383 | 0.833 |
| 35 | -1.239 | 0.128 | 0.849 |
| 36 | -1.270 | 0.351 | 0.796 |
| 37 | -0.913 | -0.182 | 0.899 |
| 38 | -0.632 | 0.013 | 0.890 |
| 39 | -0.719 | -0.323 | 0.844 |
| 40 | -1.453 | 0.013 | 0.814 |
| 41 | -0.530 | 0.018 | 0.869 |
| 42 | -0.498 | 0.266 | 0.831 |
| 43 | -0.684 | -0.156 | 0.784 |
| 44 | -0.584 | 0.369 | 0.712 |
| 45 | -0.483 | 0.373 | 0.728 |
| 46 | -0.309 | 0.493 | 0.734 |
| 47 | -0.288 | 0.350 | 0.794 |
| 48 | -0.352 | 0.377 | 0.857 |
| 49 | 0.740 | 0.039 | 0.774 |
| 50 | 0.506 | -0.009 | 0.775 |
| 51 | -0.927 | -0.100 | 0.883 |
| 52 | -0.786 | 0.187 | 0.861 |
| 53 | -0.965 | 0.169 | 0.716 |
| 54 | -1.160 | 0.478 | 0.738 |
| 55 | -1.163 | -0.252 | 0.775 |
| 56 | -0.870 | -0.118 | 0.769 |
| 57 | -0.568 | 0.215 | 0.815 |
| 58 | -0.935 | -0.020 | 0.797 |
| 59 | -0.895 | 0.000 | 0.731 |
| 60 | -0.882 | 0.030 | 0.725 |
| 61 | -0.452 | -0.201 | 0.812 |
| 62 | -0.745 | 0.116 | 0.818 |
| 63 | -1.334 | -0.137 | 0.779 |
| 64 | -1.304 | 0.054 | 0.754 |
| 65 | -0.601 | -0.431 | 0.922 |
| 66 | -0.728 | -0.311 | 0.878 |
| 67 | -0.583 | -0.519 | 0.910 |
| 68 | -0.683 | -0.186 | 0.851 |
| 69 | -0.018 | -0.231 | 0.809 |
| 70 | 0.558 | -0.113 | 0.760 |
| 71 | -1.022 | -0.184 | 0.866 |
| 72 | -1.065 | 0.032 | 0.848 |
| 73 | -0.614 | -0.522 | 0.890 |
| 74 | -0.581 | -1.088 | 0.916 |
| 75 | -1.049 | 0.091 | 0.819 |
| 76 | -1.256 | 0.617 | 0.782 |
| 77 | 0.050 | -0.856 | 0.804 |
| 78 | 0.001 | -0.783 | 0.902 |
| 79 | -0.714 | 0.176 | 0.799 |
| 80 | -0.641 | -0.086 | 0.834 |
| 81 | -1.259 | -0.045 | 0.823 |
| 82 | -1.057 | 0.280 | 0.873 |
| 83 | -0.848 | 0.307 | 0.775 |
| 84 | -0.791 | 0.392 | 0.850 |
| 85 | -1.451 | -0.131 | 0.856 |
| 86 | -1.263 | 0.272 | 0.859 |
| 87 | -0.867 | -0.112 | 0.878 |
| 88 | -0.894 | -0.124 | 0.864 |
| 89 | -0.340 | 0.343 | 0.834 |
| 90 | -0.531 | 0.236 | 0.784 |
| 91 | -0.090 | -0.395 | 0.590 |
| 92 | 0.256 | 0.019 | 0.557 |
| 93 | -0.307 | 0.445 | 0.744 |
| 94 | -0.101 | 0.652 | 0.784 |
| 95 | -0.705 | 0.187 | 0.809 |
| 96 | -0.564 | 0.413 | 0.873 |
| 97 | -1.052 | -0.140 | 0.853 |
| 98 | -0.783 | 0.161 | 0.829 |
| 99 | -0.931 | -0.077 | 0.816 |
| 100 | -0.774 | 0.280 | 0.811 |
| 101 | -0.146 | 0.499 | 0.781 |
| 102 | -0.327 | 0.538 | 0.769 |
| 103 | 0.265 | 0.176 | 0.797 |
| 104 | 0.373 | 0.137 | 0.773 |
| 105 | -0.051 | -0.196 | 0.782 |
| 106 | -0.216 | -0.002 | 0.776 |
| 107 | -0.289 | -0.068 | 0.788 |
| 108 | 0.190 | 0.239 | 0.762 |
| 109 | -0.148 | -0.089 | 0.736 |
| 110 | -0.177 | 0.202 | 0.787 |
| 111 | 0.968 | -0.155 | 0.642 |
| 112 | 0.929 | 0.031 | 0.622 |
| 113 | -0.042 | -0.005 | 0.670 |
| 114 | 0.004 | 0.188 | 0.728 |
| 115 | 1.381 | -0.692 | 0.492 |
| 116 | 1.168 | -0.401 | 0.280 |
| 117 | 0.825 | -0.497 | 0.522 |
| 118 | 0.992 | -0.583 | 0.457 |
| 119 | 0.668 | -0.743 | 0.765 |
| 120 | 0.698 | -0.776 | 0.671 |
| 121 | -0.591 | -0.188 | 0.872 |
| 122 | -0.610 | -0.508 | 0.872 |
| 123 | -0.667 | -0.106 | 0.882 |
| 124 | -0.969 | -0.376 | 0.837 |
| 125 | -1.152 | -0.184 | 0.859 |
| 126 | -1.284 | -0.192 | 0.868 |
| 127 | -1.089 | -0.228 | 0.890 |
| 128 | -0.799 | 0.107 | 0.917 |
| 129 | -0.955 | -0.392 | 0.903 |
| 130 | -1.035 | -0.562 | 0.910 |
| 131 | -0.643 | -0.011 | 0.879 |
| 132 | -0.916 | -0.300 | 0.868 |
| 133 | -0.930 | -0.135 | 0.888 |
| 134 | -0.660 | -0.051 | 0.908 |
| 135 | -1.000 | 0.058 | 0.893 |
| 136 | -1.298 | 0.098 | 0.876 |
| 137 | -1.230 | -0.402 | 0.921 |
| 138 | -1.037 | -0.119 | 0.892 |
| 139 | -1.149 | -0.429 | 0.877 |
| 140 | -0.970 | -0.310 | 0.876 |
| 141 | -1.470 | -0.275 | 0.864 |
| 142 | -1.480 | 0.057 | 0.838 |
| 143 | -1.028 | -0.176 | 0.897 |
| 144 | -1.121 | -0.199 | 0.902 |
| 145 | -1.148 | -0.203 | 0.849 |
| 146 | -1.144 | -0.215 | 0.800 |
| 147 | -0.896 | -0.667 | 0.926 |
| 148 | -0.623 | -0.219 | 0.914 |
| 149 | -0.638 | -0.304 | 0.910 |
| 150 | -0.736 | -0.390 | 0.918 |
| 151 | -0.617 | -0.127 | 0.914 |
| 152 | -0.880 | 0.028 | 0.922 |
| 153 | -1.025 | -0.632 | 0.907 |
| 154 | -0.840 | -0.411 | 0.916 |
| 155 | -1.164 | 0.030 | 0.745 |
| 156 | -1.208 | 0.227 | 0.742 |
| 157 | -0.662 | -0.210 | 0.806 |
| 158 | -0.439 | 0.070 | 0.805 |
| 159 | -1.079 | -0.301 | 0.859 |
| 160 | -1.096 | -0.193 | 0.801 |
| 161 | -0.883 | -0.057 | 0.850 |
| 162 | -0.872 | 0.121 | 0.820 |
| 163 | -0.741 | -0.105 | 0.766 |
| 164 | -0.912 | -0.367 | 0.845 |
| 165 | -0.493 | -0.807 | 0.700 |
| 166 | -0.017 | -0.438 | 0.639 |
| 167 | -0.741 | -0.272 | 0.822 |
| 168 | -0.563 | -0.040 | 0.861 |
| 169 | -0.709 | -0.956 | 0.782 |
| 170 | -0.264 | -0.460 | 0.817 |
| 171 | -0.381 | -0.091 | 0.795 |
| 172 | -0.380 | 0.033 | 0.797 |
| 173 | -0.729 | -0.486 | 0.722 |
| 174 | -0.121 | 0.047 | 0.798 |
| 175 | -0.831 | -0.457 | 0.819 |
| 176 | -0.897 | -0.575 | 0.892 |
| 177 | -0.915 | -1.045 | 0.931 |
| 178 | -0.758 | -1.126 | 0.925 |
| 179 | -1.044 | -0.744 | 0.869 |
| 180 | -0.877 | -0.386 | 0.816 |
| 181 | 0.278 | -0.486 | 0.845 |
| 182 | -0.517 | -0.678 | 0.847 |
| 183 | -1.227 | -0.682 | 0.846 |
| 184 | -1.093 | -0.324 | 0.787 |
| 185 | -0.739 | -0.450 | 0.869 |
| 186 | -0.905 | -0.324 | 0.854 |
| 187 | -0.288 | -0.315 | 0.791 |
| 188 | -0.570 | -0.291 | 0.789 |
| 189 | 0.438 | -0.074 | 0.702 |
| 190 | 0.578 | 0.009 | 0.555 |
| 191 | -0.113 | 0.082 | 0.871 |
| 192 | -0.162 | 0.208 | 0.865 |
| 193 | 0.019 | -0.043 | 0.659 |
| 194 | -0.044 | 0.322 | 0.793 |
| 195 | 0.185 | -0.187 | 0.867 |
| 196 | -0.259 | -0.021 | 0.772 |
| 197 | -0.518 | 0.037 | 0.906 |
| 198 | -0.831 | -0.039 | 0.884 |
| 199 | -1.019 | 0.322 | 0.902 |
| 200 | -0.858 | 0.409 | 0.860 |
| 201 | -1.272 | 0.078 | 0.910 |
| 202 | -1.100 | 0.331 | 0.854 |
| 203 | -1.164 | 0.521 | 0.792 |
| 204 | -1.028 | 0.882 | 0.785 |
| 205 | -0.174 | 0.298 | 0.590 |
| 206 | 0.182 | 0.264 | 0.617 |
| 207 | -0.487 | 0.368 | 0.893 |
| 208 | -0.842 | 0.418 | 0.872 |
| 209 | -0.930 | -0.033 | 0.878 |
| 210 | -0.735 | 0.129 | 0.908 |
| 211 | 0.831 | -1.154 | 0.448 |
| 212 | 0.893 | -0.940 | 0.482 |
| 213 | -0.359 | -0.023 | 0.677 |
| 214 | 0.019 | -0.120 | 0.653 |
| 215 | 1.060 | -0.974 | 0.672 |
| 216 | 0.804 | -0.782 | 0.718 |
| 217 | 0.626 | -1.120 | 0.697 |
| 218 | 0.755 | -0.761 | 0.680 |
| 219 | -0.443 | -0.079 | 0.892 |
| 220 | -0.782 | 0.430 | 0.737 |
| 221 | -0.666 | 0.116 | 0.721 |
| 222 | -0.577 | 0.058 | 0.799 |
| 223 | 0.111 | 0.089 | 0.658 |
| 224 | -0.099 | -0.179 | 0.708 |
| 225 | -0.439 | 0.705 | 0.750 |
| 226 | -0.489 | 0.743 | 0.738 |
| 227 | -0.559 | -0.702 | 0.956 |
| 228 | -0.869 | -0.974 | 0.928 |
| 229 | -0.574 | 0.700 | 0.757 |
| 230 | -0.431 | 0.847 | 0.713 |
| 231 | -0.450 | -0.280 | 0.899 |
| 232 | -0.791 | 0.040 | 0.729 |
| 233 | -0.548 | 0.338 | 0.750 |
| 234 | -0.793 | 0.681 | 0.492 |
| 235 | -0.678 | 0.613 | 0.568 |
| 236 | -0.604 | 0.962 | 0.499 |
| 237 | 0.121 | -1.049 | 0.927 |
| 238 | -0.356 | -1.324 | 0.942 |
| 239 | -0.668 | 0.680 | 0.615 |
| 240 | -0.682 | -0.194 | 0.729 |
| 241 | -0.139 | -0.600 | 0.814 |
| 242 | -0.418 | -1.174 | 0.775 |
| 243 | -0.084 | -0.669 | 0.849 |
| 244 | -0.317 | -0.671 | 0.787 |
| 245 | -0.837 | 0.772 | 0.562 |
| 246 | -0.824 | 0.660 | 0.512 |
